# Ectopic Pelvic Kidney Mimicking Sacral Metastasis on Post-Therapy Iodine-131 Scan of a Thyroid Cancer Patient

**DOI:** 10.4274/mirt.75436

**Published:** 2017-02-01

**Authors:** Selin Soyluoğlu Demir, Gül Ege Aktaş, Ahmet Polat, Ali Sarıkaya

**Affiliations:** 1 Trakya University Faculty of Medicine, Department of Nuclear Medicine, Edirne, Turkey; 2 Sultan 1. Murat State Hospital, Clinic of Radiology, Edirne, Turkey

**Keywords:** thyroid cancer, ectopic kidney, Iodine-131, bone scan, false-positive

## Abstract

A 25-year-old woman had total thyroidectomy and iodine-131 ablation therapy for papillary thyroid carcinoma. Whole body imaging on the 7^th^ day of therapeutic activity demonstrated radioiodine uptake in the remnant tissue and intense heterogeneous uptake at the sacral region prominently in the posterior image. Initial interpretation was suspicious for sacral metastasis. Technetium-99m-methylene diphosphonate bone scan demonstrated normal bone uptake and the absence of left kidney. On blood-pool phase of bone scan, the absence of left renal activity and an extra area of uptake in the sacral region suggestive of pelvic kidney were noticed. Magnetic resonance imaging scan confirmed the ectopic pelvic kidney overlying the sacrum.

## Abstract (tr)

Papiller tiroid karsinomu tanısı alan 25 yaşında kadın hastaya total tiroidektomi ve sonrasında iyot-131 ablasyon tedavisi uygulandı. Terapötik aktivite sonrası 7. gün yapılan tüm vücut görüntülemede tiroid lojunda bakiye dokuya ait yoğun aktivite tutulumu ile sakral bölgede posterior imajlarda daha belirgin olmak üzere yoğun, heterojen bir aktivite tutulum alanı saptandı. Görünüm sakral metastaz şüphesi uyandırmaktaydı. Teknesyum-99m-metilendifosfonat kemik sintigrafisinde kemiklerde normal dağılım izlenirken, sol böbreğin normal lokalizasyonunda bulunmadığı görüldü. Kemik sintigrafisi kan havuzu fazı incelendiğinde, sol böbrek aktivitesi yerinde izlenmezken, sakral bölgede pelvik böbreğe ait olabilecek ekstra bir aktivite tutulum alanı saptandı. Yapılan manyetik resonans görüntüleme ile sakrum önünde lokalize ektopik pelvik böbrek doğrulandı.

## INTRODUCTION

Differentiated thyroid cancer is a possibly curable cancer that is associated with low mortality rates. It is usually managed by total thyroidectomy followed by iodine-131 (I-131) ablation of remnant thyroid tissue. Nevertheless, 1-3% of patients may have distant metastases at initial diagnosis, and another 7-23% may develop distant metastases during disease course. The distant metastases, particularly bone metastases, increase mortality rate and decrease quality of life. Radioiodine has been used for decades for the diagnosis and treatment of patients with papillary or follicular thyroid carcinoma, and patients are mainly followed-up with whole-body I-131 scintigraphy (WBS) and thyroglobulin levels. However, it is important to identify false-positive sites to avoid unnecessary ablation therapy. Herein we report a case of a demonstrative example of pelvic kidney mimicking sacral metastasis on I-131 WBS.

## CASE REPORT

**Figure 1:**
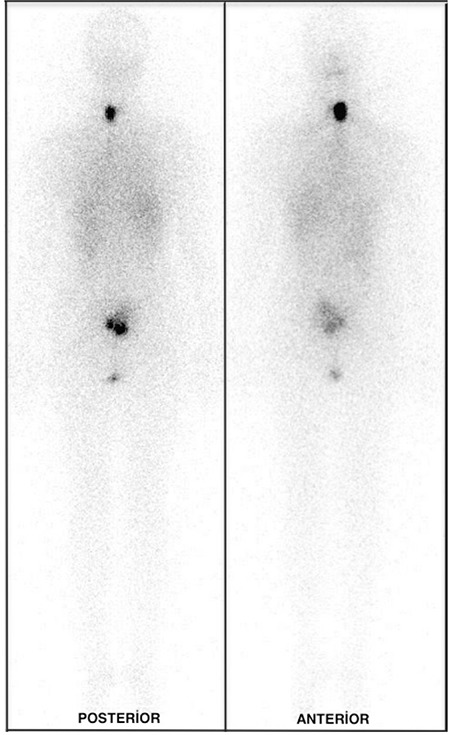
A whole-body scintigraphy performed 7 days after administration of iodine-131 showed intense uptake corresponding to thyroid remnant tissue and a heterogeneous radioiodine uptake in the sacral region, prominently in the posterior image

**Figure 2:**
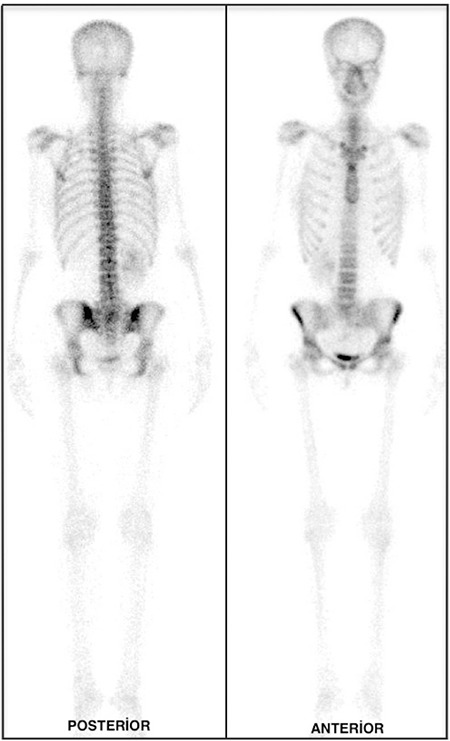
Osteoblastic phase of methylene diphosphonate bone scan demonstrated normal bone uptake and absence of the left kidney

**Figure 3:**
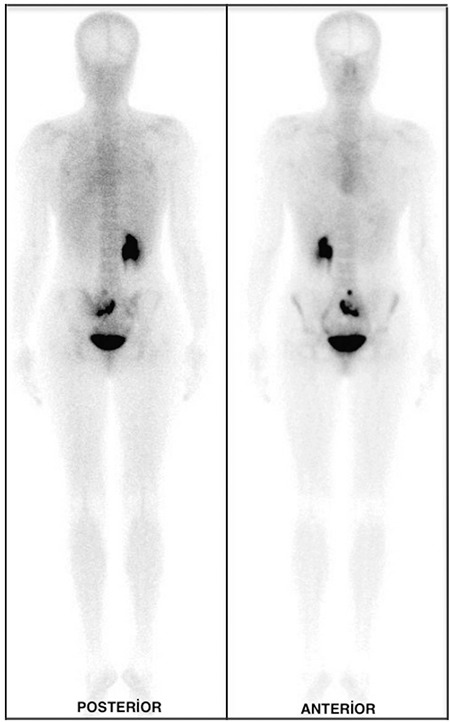
The absence of left renal activity and an extra focus of uptake in the sacral region due to pelvic kidney was seen on blood-pool phase

**Figure 4:**
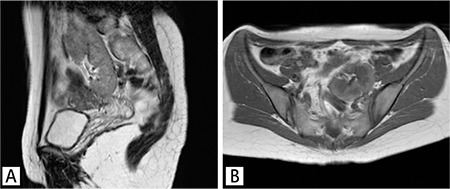
Axial T1-weighted (A) and coronal T2-weighted (B) magnetic resonance imaging demonstrated an ectopic kidney located in the left posterior pelvic region overlying the sacrum with an anteriorly rotated renal pelvis

A 25-year-old woman underwent total thyroidectomy and central neck lymph node dissection with histopathologic diagnosis of papillary thyroid carcinoma. Three months after surgery, 5550 MBq (150 mCi) of I-131 was administered for thyroid remnant ablation. Post treatment WBS on the 7^th^ day of treatment demonstrated intense uptake corresponding to thyroid remnant tissue and a heterogeneous radioiodine uptake in the sacral region, prominently in the posterior image (Figure 1). Initial interpretation was suspicious for sacral metastasis. No other abnormal focus was determined elsewhere in the whole body. The serum thyroglobulin level was 13.6 ng/ml and anti-thyroglobulin was 196.4 U/ml, while thyroid stimulating hormone value was 123.9 mIU/ml. Because of the patient’s pelvic pain complaint and intermediate-high risk according to thyroglobulin level, a technetium-99m-methylene diphosphonate (MDP) bone scan was also performed. The osteoblastic phase of the bone scan demonstrated normal bone uptake and absence of the left kidney (Figure 2). We routinely perform 2-5-minute whole-body blood-pool imaging in our department, especially in case of whole-body tumor and metastasis evaluation with MDP bone scan. In some cases, the normal kidneys and urinary system can be cleared thoroughly in MDP bone scan and ectopic kidneys can be confusing. When the blood-pool phase was analyzed, absence of the left renal activity and an extra focus of uptake in the sacral region due to pelvic kidney was noticed (Figure 3). Magnetic resonance imaging (MRI) of patient confirmed the ectopic kidney located in the left posterior pelvic region overlying the sacrum with an anteriorly rotated renal pelvis (Figure 4).

## LITERATURE REVIEW AND DISCUSSION

Radioiodine is a sensitive marker for diagnosing metastases of differentiated papillary or follicular thyroid carcinoma; however, is not specific for thyroid tissue. It can also be seen in normal tissue, including salivary glands, thymus, breast, liver, and gastrointestinal tract, or in benign or malignant non-thyroidal diseases, such as sinusitis, esophagus and gastric pathologies, pulmonary diseases, cysts and traumatic lesions, which could be mistaken for thyroid cancer metastases (1) and lead to unsuitable therapy, such as unnecessary reoperations and/or administration of repeated doses of I-131.

More than 90% of iodide is excreted from the body by the kidneys. As a result, physiologic urine activity or urine retention in dilated renal collecting systems can be seen on I-131 WBS. Ureteral or bladder diverticulum and renal cysts have been reported to cause false-positive findings mimicking abdominal and pelvic region metastases (2,3,4). Ectopic kidneys can also be a cause of false-positive WBS.

Ectopic kidneys are due to developmental anomalies and may be located at the pelvic, iliac fossa or lumbar region, anywhere along the path of their usual ascent. If the kidney stays in the pelvic fossa during the ascending process, it is called a pelvic kidney. This anomaly can be unilateral or bilateral. The incidence of ectopic pelvic kidney is reported as 1:2100-1:3000 in autopsy series (5).

Ectopic kidneys are mostly asymptomatic. Hydronephrosis is seen in half of the patients, due to malrotation of the kidney and anteriorly placed renal pelvis leading to impaired urinary drainage (6). In our case, the pelvic kidney was located in the left posterior pelvic region overlying the sacrum with an anteriorly rotated renal pelvis (Figure 4, A axial; B sagittal).

As an additional finding, MRI reported that the pelvic kidney was adjacent to the left ovary. Because of the hydronephrosis seen quite often in pelvic kidneys, retention of high dose I-131 activity in this region may increase dosimetry of the ovarian tissue. Therefore, in case of an ectopic kidney detected prior to ablation treatment, precautions such as hydration and diuretic administration can be implemented.

Although ectopic kidney is a well-known finding, there are only few demonstrative cases showing ectopic kidney as a false-positive area for I-131 WBS (7,8), and few reports for MDP bone scan or other diagnostic modalities (9,10,11). Our case is a demonstrative example of pelvic kidney mimicking sacral metastases on I-131 WBS.
